# Cellular and animal models to investigate pathogenesis of amyloid aggregation in neurodegenerative diseases

**DOI:** 10.52601/bpr.2022.210033

**Published:** 2022-02-28

**Authors:** Houfang Long, Shuyi Zeng, Dan Li

**Affiliations:** 1 Interdisciplinary Research Center on Biology and Chemistry, Shanghai Institute of Organic Chemistry, Chinese Academy of Sciences, Shanghai 201210, China; 2 University of Chinese Academy of Sciences, Beijing 100049, China; 3 Bio-X Institutes, Key Laboratory for the Genetics of Developmental and Neuropsychiatric Disorders, Ministry of Education, Shanghai Jiao Tong University, Shanghai 200030, China; 4 Bio-X-Renji Hospital Research Center, Renji Hospital, School of Medicine, Shanghai Jiao Tong University, Shanghai 200240, China; 5 Zhangjiang Institute for Advanced Study, Shanghai Jiao Tong University, Shanghai 200240, China

**Keywords:** Amyloid fibril, Primary neuron, Mouse model, Parkinson’s disease, α-Synuclein

## Abstract

Abnormal aggregation of amyloid proteins, *e*.*g*. amyloid β (Aβ), Tau and α-synuclein (α-syn), is closely associated with a variety of neurodegenerative diseases such as Alzheimer’s disease (AD) and Parkinson’s disease (PD). Cellular and animal models are useful to explore the neuropathology of amyloid aggregates in disease initiation and progression. In this protocol, we describe detailed procedures for how to establish neuronal and PD mouse models to evaluate amyloid pathologies including self-propagation, cell-to-cell transmission, neurotoxicity, and impact on mouse motor and cognitive functions. We use α-syn, a key pathogenic protein in PD, as an example to demonstrate the application of the protocol, while it can be used to investigate the pathologies of other amyloid proteins as well. The established disease models are also useful to assess the activities of drug candidates for therapeutics of neurodegenerative diseases.

## INTRODUCTION

Amyloid protein aggregation is a common pathological hallmark of neurodegenerative diseases, which plays essential roles in the initiation and progression of the diseases (Arai* et al.* 2006; Goedert* et al.* 1988; Murphy and LeVine 2010; Spillantini* et al.* 1997). For example, α-synuclein (α-syn) forms amyloid fibrils depositing into intraneuronal Lewy bodies/Lewy neurites (LBs/LNs) that serve as a histological hallmark of Parkinson’s disease (PD) (Baba* et al.* 1998; Spillantini* et al.* 1997). Moreover, during the progression of PD, α-syn fibrils self-propagate and spread via cell-to-cell transmission across PD patients’ brains (Luk* et al.* 2012), which cause degeneration of midbrain dopamine (DA) neurons in the substantia nigra pars compacta (SNpc) (Damier* et al.* 1999) leading to bradykinesia, tremor, and postural instability (Luk* et al.* 2012). Studies have shown that α-syn preformed fibrils (PFFs) but not monomers induce the formation of LBs/LNs-like inclusions by recruiting and converting endogenous soluble monomeric α-syn protein into insoluble aggregates in primary neurons (Luk* et al.* 2009; Volpicelli-Daley* et al.* 2014). Moreover, administration of synthetic α-syn PFFs into mouse brain through stereotaxic injection leads to LBs/LNs formation in multiple brain regions and impairment of motor function (Zhang* et al.* 2019). Based on recent advances in cellular and animal model studies of amyloid fibrils (Long* et al.* 2021; Volpicelli-Daley* et al.* 2014; Zhang* et al.* 2019), we describe a series of assays to study α-syn fibril pathologies in cells and *in vivo*, which may help to understand the formation and the neurological effects of α-syn fibrils and further develop therapeutic drugs to cease PD progression.

## Development of the protocol

The protocol is mainly composed of four parts, including α-syn PFFs preparation, cell viability assay, primary neuron model, and PD mouse model. The protocol provides a systematic pipeline to study the pathological properties of different α-syn fibril strains. For the preparation of α-syn PFFs, the concentration of fibrils needs to be directly and accurately measured since different amyloid proteins exhibit distinct fibril converting rates. Next, the propagation of α-syn PFFs in primary neurons is investigated by monitoring the neuronal aggregation of α-syn at different time points. α-Syn pathology is further evaluated in a PD-related mouse model induced by injecting exogenous α-syn PFFs. In sum, we provide a detailed protocol that is useful to study the pathology of α-syn amyloid fibrils in cells and *in vivo*.

## Applications and advantages of the protocol

This is a systematic protocol for evaluating the pathological amyloid fibrils both in cells and* in vivo*. This protocol is not limited to α-syn study, but also can be applied to investigate neuropathological activities of other amyloid proteins such as Tau, TDP-43 (TAR DNA-binding protein 43kDa) and FUS (fused in sarcoma). This protocol describes the preparation and characterization of amyloid fibrils, measurement of the cytotoxicity of fibrils, endogenous aggregation and propagation induced by fibrils in primary neurons, and assessment of the fibril pathology in disease-related mouse models. These models are well-established with defined disease-related phenotypes and relatively easy for setup in biological laboratories following the detailed instructions described below. Moreover, these models provide important disease-related cellular and animal models to evaluate antibodies and inhibitors screened from *in*-*vitro* assay for the drug discovery of neurodegenerative diseases.

## Limitations of the protocol

1 As for the mouse model, the stereotaxic injection site of fibrils in the brain, dorsal striatum (dSTR), described in this protocol is the most suitable for α-syn fibrils. For other pathological amyloid fibrils such as Tau fibrils and TDP-43 fibrils, different injection sites may need to be explored to better mimic their pathologies in diseased brains.

2 As for the behavioral tests, mice are easily affected by the environment and the tester. Consistency should be carefully controlled in experiments.

3 During the process of transcranial perfusion and frozen sectioning, man-made interferences are inevitable and should be considered.

4 Both the integrity and symmetry of brain slices influence the quality of immunofluorescence imaging.

5 Additional behavioral test assays may need to be performed including water maze, shuttle box experiment and balance beam experiment to evaluate the cognitive and motor dysfunction of the diseased mice.

## Overview of the protocol

Firstly, the cytotoxicity of α-syn PFFs is examined by using SH-SY5Y neuroblastoma cell line with CCK-8 kit. Next, exogenous α-syn PFFs inducing endogenous α-syn aggregates are studied in primary neurons, which is characterized by immunofluorescence staining. Then, mice are inoculated with α-syn PFFs leading to motor deficit and endogenous α-syn amyloid aggregates *in vivo*. Together, the protocol is applied to test the neurotoxicity and aggregation of α-syn fibrils both in cells and *in vivo*.

## SUMMARIZED PROCEDURE

### α-Syn fibril preparation

1 α-Syn protein is purified and incubated to form fibrils. Fibril formation is characterized by transmission electron microscope (TEM).

2 To measure the yield of α-syn fibrils, centrifuge the fibril sample at 14,500 r/min, 25 °C for 40–50 min. Subtract the amount of residual soluble α-syn from the total amount of α-syn monomers.

3 α-Syn fibrils are sonicated into short α-syn PFFs and stored at –80 °C.

### Cellular assay

1 Culture and plate SH-SY5Y cells for α-syn PFFs treatment. Cell viability is assessed by using the CCK-8 kit.

2 Preparation for primary neuron culturing, such as plate coating, medium setup, papain activation, and so on.

3 Dissect the cerebral cortex of E16–E18 rat embryos, and settle them in HBSS buffer on ice.

4 Digest the brain tissue with activated papain and DNase for 30 min. And then filter the brain tissue after digestion through a 40-mm nylon mesh cell strainer.

5 Resuspend the cell pellet and perform cell counting. Plate cells as 10–15 × 10^4^ cells/well and put plates into a culture incubator.

6 At the 8^th^ day* in vitro* (DIV), treat neurons with α-syn PFFs, incubate these neurons for desired days, and collect samples.

7 To stain primary neurons, we fix them with 4% paraformaldehyde (PFA) after rinsing with phosphate buffered saline (PBS). Then permeabilize neurons and block them.

8 Incubate coverslips with the primary antibody at 4 °C overnight followed by washing with PBST (0.1% Triton X-100 in PBS).

9 Secondary antibodies are incubated with neurons at room temperature (RT) for 1 h in a dark room followed by washing with PBST.

10 Mount coverslips on glass slides with the medium.

### Disease-related mouse model

1 For stereotaxic injection, first weigh mice that are at an age of eight weeks.

2 Fix mice gently on the stereotaxic apparatus after anesthesia.

3 Shave the hair on the top of the mouse’s head and then cut the scalp with scissors to expose the skull.

4 Position the brain area to be injected (dSTR for α-syn) and drill a hole at the target region.

5 Inject α-syn PFFs with a micro syringe. The required amount of PFFs for injection is calculated according to the weight of the mouse, 0.2 μg/g is suggested.

6 At 3-month post injection (mpi), behavioral tests are performed. Before the open field test (OFT), mice are handled for 3 d.

7 After OFT, mice need to rest for one day.

8 Then rotarod training and tests are performed.

9 After the mice rest for one day, pole tests are performed.

10 Then the mice are sacrificed for immunofluorescence staining experiments. Mice brains go through dehydration for three times with sucrose dissolved in PBS after perfusion and fixation.

11 Each brain tissue is serially sectioned into 30-μm slices with a cryostat microtome.

12 Brain slices are blocked with 10% goat serum and stained with primary antibodies and fluorescent secondary antibodies.

13 Mounting slices on glass slides with the medium.

14 Spinning disk fluorescence confocal microscope is required for imaging.

## EXPERIMENTAL DESIGN

### α-Syn PFFs preparation

After the purification of the monomeric α-syn protein, filter the protein with a 0.22-μm centrifugal filter. Then α-syn fibril is prepared as previously reported (Li* et al.* 2018). Calculate the yield of α-syn fibril by subtracting the amount of residual soluble α-syn after pelleting the fibrils with the total amount of α-syn monomers. Then α-syn PFFs are produced by sonication with a probe tip sonicator. The morphologies of α-syn fibrils and PFFs are characterized by TEM. The PFF stock solution is stored at –80 °C.

### Cell viability assay

Cell viability is measured by using a CCK-8 kit. SH-SY5Y cells are cultured and plated in a 96-well plate (6,000 cells/well, 100 μL/well). Then α-syn PFFs are applied to treat SH-SY5Y cells with final concentrations of 0, 0.01, 0.1 and 1 µmol/L (equivalent to monomer concentration) for 24 h. Further test the cell viability with CCK-8 kit following the manufacturer’s protocol. Briefly, add 10 μL CCK-8 solution to each well, incubate the plate for 1–4 h in darkness. Finally, the absorbance of each well is measured at 450 nm.

### Primary neuron culture and cell transmission assay

Primary cortical neurons are cultured from E16-E18 embryos of pregnant Sprague-Dawley (SD) rats. Dissociated cortical neurons are cultured in a 24-well plate at a density of 10–20 × 10^4^ cells/well. Primary neurons are treated with α-syn PFFs at 8 DIV with a final concentration of 100 nmol/L. Neurons are harvested for immunofluorescence staining at different time points, such as 22 DIV, 26 DIV, 30 DIV, and so on.

### Stereotaxic injection

Eight-week old C57BL/6J male mice are anesthetized with 0.5% isoflurane mixed with 1% O_2_. α-Syn PFFs are stereotaxically injected into dSTR (coordination: +0.2 mm to bregma (B), ±2.0 mm from midline, −2.6 mm from dura.) of both hemispheres at a dose of 0.2 μg/g (body weight). Control mice are bilaterally injected with sterile PBS 1 µL/10 g (body weight).

### Behavioral tests

At 1 mpi, 3 mpi, 6 mpi and so on, all mice receive behavioral training and tests. The behavioral tests include open field test, rotarod test and pole test. All tests are carried out between Zeitgeber time 4–8 (4–8 h post light on). Mice are transferred to the behavioral room at least 1 h prior to the test for sufficient habituation to the testing environment.

### Immunofluorescence staining

Mice are anesthetized with isoflurane vapor and transcranially perfused. The brains are fixed by 4% PFA and dehydrated with sucrose solution. Then the brains are sectioned into 30-μm coronal slices with cryostat microtome. Right before staining, brain slices are first blocked and then incubated with primary antibody at 4 °C overnight. The slices are further incubated in blocking buffer containing secondary antibody for 2 h at RT after being washed three times with PBS. Lastly, slices are mounted on glass slides with the mounting medium.

## PROCEDURE

### α-Syn PFFs preparation [TIMING 8 d]

1 α-Syn protein is purified as previously described (Li* et al.* 2018) and is filtered with the 0.22-μm centrifugal filter to remove precipitates.

2 To incubate fibril, α-syn protein (200 µmol/L, 200 µL in 50 mmol/L Tris, pH 7.5, 150 mmol/L KCl) in a 1.5-mL microtube is placed in the ThermoMixer, at 37 °C with constant agitation (900 r/min) for 7 d.

3 After the maturation of α-syn fibril, the morphology of fibrils is characterized by TEM.

4 α-Syn fibril is centrifuged at 14,500 r/min, 25 °C for 40–50 min. Then remove the supernatant (volume of supernatant, *V*_s_) and measure the concentration of residual soluble α-syn (*C*_s_). The amount of α-syn fibril is calibrated by subtracting the amount of residual soluble α-syn (*C*_s_ × *V*_s_) with the total amount of α-syn monomer (*C*_t_ × *V*_t_). The equation for the concentration of α-syn fibril (*C*_p_) is *C*_p_= (*C*_t_ × *V*_t_ – *C*_s_ × *V*_s_) / (*V*_t_ – *V*_s_).

5 α-Syn fibril is resuspended with sterile PBS to a final concentration of 100 µmol/L, and sonicated into α-syn PFFs with a probe tip sonicator, with 20% power, 1 s on, 1 s off, total time 30 s. The morphology of PFFs is characterized by TEM.

**[TIP]** Divide PFFs into aliquots and store them at –80 °C to avoid multiple freeze-thaw cycles.

### Cell viability test [TIMING 2–5 d]

6 SH-SY5Y cells culture. Maintain cells with DMEM medium containing 10% fetal bovine serum (FBS) and 1% penicillin-streptomycin (PS) in the 37 °C, 5% CO_2_ cell culture incubator.

7 Plating. Trypsin-digested SH-SY5Y cells (6,000 cells/well) are plated to a 96-well plate with a volume of 90 µL in each well. Setting triplicate wells of each sample group.

8 Treatment. Cells are treated with 10 µL α-syn PFFs at final concentrations of 0, 0.01, 0.1 and 1 µmol/L. Incubate for a desired period (*e*.*g*., 24 h, 48 h, 96 h and so on) in a culture incubator.

**[TIP]** Sample concentration and treatment time can be adjusted according to different amyloid fibril samples.

9 The cell viability is measured by using a CCK-8 kit following the manufacturer’s protocol. Briefly, add 10 μL of CCK-8 solution to each sample well and mix by gentle tapping. And incubate the plate at 37 °C for 1–4 h protected from light. Then measure the absorbance at 450 nm. Lastly, calculate the cell viability according to the equation: cell viability (%) = [A_(treatment)_ – A_(blank)_] / [A_(0)_ – A_(blank)_] × 100.

**[TIP]** Avoid bubbles in wells before plate reading.

**[?TROUBLESHOOTING]**

### Primary neuron culture and PFF propagation assay

#### Primary neuron culture [TIMING 5 h]

10 Preparation

(A) Coating coverslips with Poly-D-Lysine (PDL). Soak the coverslips in 75% alcohol, then dip them in absolute ethanol, and quickly burn and dry them with an alcohol lamp. Add 350 µL PDL buffer (0.01 mg/mL, diluted with borate buffer) to each well where the coverslips are placed, and place plates in a 37 °C cell incubator overnight.

**[TIP]** Slides are fragile and should be placed in the central eight wells (middle-area) of the 24-well plate.

**[?TROUBLESHOOTING]**

(B) Washing. Wash plates with sterilized H_2_O three times the next day, dry and irradiate with ultraviolet (UV) in ultra clean desk.

(C) Medium setup. Calculate the amount of plating medium and Neurobasal medium required (0.5 mL plating medium for each well of 24-well plate and 1 mL Neurobasal medium for each well of 24-well plate) and prepare these media.

(D) Papain activation. Papain is activated by adding papain buffer. Shake well to translucent.

(E) HBSS setup. Add 5–10 mL 1mol/L HEPES to 500 mL HBSS. Take out 100 mL and put on ice for later use.

(F) Dissecting instrument preparation. Disinfect dissecting instruments with 75% alcohol before using.

11 Anatomy. Pregnant rat is anesthetized with isoflurane and sacrificed by cervical dislocation. After dissecting the rat, E16–E18 embryos are taken out, the cerebral cortex of the embryos is stripped, and then the meninges are removed. Then put the cerebral cortex into a 15-mL centrifuge tube containing HBSS buffer on ice.

12 Washing. Remove HBSS buffer from the centrifuge tube slowly, and rinse for five times, 10 mL HBSS/time.

**[TIP]** Avoid sucking the cells away.

13 Digestion. Transfer the tissue to a 1.5-mL microtube containing 400 µL activated papain and 50 µL DNase. Cut the brain tissue with scissors. Then place the microtube in a 37 °C incubator for 30 min, and gently shake it every 5 min.

**[TIP]** During the digestion process, seal the edge wells of the 24-well plate with sterilized H_2_O, 1.0 mL/well.

**[?TROUBLESHOOTING]**

14 Filtering. Filter the brain tissue after digestion through a 40-mm nylon mesh cell strainer. Rinse the strainer with a 15 mL plating medium. Centrifuge at 900 r/min for 5 min, discard the supernatant and resuspend the cell pellet with 2 mL medium gently.

15 Plating. Transfer the cell resuspension solution to a 50-mL centrifuge tube, then add plating medium to 20 mL. Perform cell counting and plate neurons as 10–15 × 10^4^ cells/well, 0.5 mL medium/well.

16 Changing medium. After plating for 1–2 h, change the plating medium to Neurobasal medium completely, 1 mL/well. Place them in the culture incubator.

#### PFF propagation assay [TIMING 35 d]

17 Neuron treatment. At 8 DIV post neuron plating, add α-syn PFFs to medium at a final concentration of 100 nmol/L. Set an equivalent volume of PBS as a control.

**[?TROUBLESHOOTING]**

18 Cell collection. Incubate the neurons for a further 14–22 d. Collect the cell samples at 22, 26 and 30 DIV.

19 Immunofluorescence staining [TIMING 3 d]

(A) Remove the medium from coverslips and rinse wells with PBS.

**[TIP]** The operation should be as gentle as possible to prevent the cells from being washed away.

**[?TROUBLESHOOTING]**

(B) Fixation. Apply fixation solution (4% PFA, 4% sucrose in PBS) to neurons. Incubate for 10 min, at RT.

(C) Washing. Wash coverslips for three times with PBS gently, 10 min/time.

(D) Permeabilization. Permeabilize neurons with 0.15% Triton X-100 in PBS, 15 min, at RT.

(E) Blocking. Remove permeabilization solution and block neurons with 5% bovine serum albumins (BSA) in PBS, 30 min, at RT.

**[TIP]** The plates can be kept at 4 °C for one week.

(F) Primary antibody incubation. Incubate neurons with primary antibodies (anti-α-synuclein (phosphor-S129) antibody, ab51253, 1:1000; anti-MAP2 antibody, ab5392, 1:1000; diluted with 5% BSA in PBS) at 4 °C overnight or 1 h, at RT with shaking.

(G) Washing. Rinse plates with PBST (0.1% Triton X-100 in PBS), 10 min/time, three times, shaking.

(H) Secondary antibody incubation. Incubate neurons with secondary antibodies (goat anti-rabbit IgG Alexa Fluor 568 (1:1000, abcam, ab175471) and goat anti-chicken Alexa Fluor 488 (1:1000, Thermo Fisher, A-11039) for 1 h at RT in darkness, shaking.

(I) Mounting coverslips. Coverslips are mounted on glass slides with ProLong™ Gold Antifade Mountant with DAPI, kept at 4 °C.

**[TIP]** Be careful to avoid air bubbles when mounting the coverslips. After mounting, put it at RT until the mounting medium is dry, and keep it at 4 °C.

(J) Visualize α-syn aggregation in primary neurons with a confocal fluorescence microscope.

### PD mouse modeling [TIMING 35 d]

#### Stereotaxic injection [TIMING 1–2 h/mouse]

20 Weighing. Put the mice on the weighing scale, and record their weight.

21 Fixed position. Eight-week-old male mice are under anesthesia. Then fix mice softly on the stereotaxic apparatus. The incisor of the mouse is snapped into the front end of the animal adapter. And the ear rods are fixed near the ear socket of mice. The mice are continuously anesthetized with 0.5% isoflurane mixed with 1% O_2._

**[TIP]** If ear rods are fixed on the respiratory center, the lower limbs of mice would jump up. During surgery, the anesthetized mouse is placed on a heating pad to maintain body temperature at 37 °C.

22 Shaving. Use a razor to shave the hair on the top of the mouse's head, apply depilatory cream for a short while, and then wipe the hair with a cotton swab.

**[TIP]** While waiting for the depilatory cream to work, apply erythromycin ointment on the eyes of mice. Put ear studs on the mice. Change the cranial drill bit.

23 Anatomy. Lift the scalp with tweezers and cut it vertically with scissors (from the eyes to the base of the ears, around 1 cm long). Corrode the meninges with hydrogen peroxide (H_2_O_2_) for 10 s, and then wipe clean with a cotton swab dipped in saline.

**[TIP]** The cranium is fully exposed, and Bregma and Lambda are clearly shown under the effect of H_2_O_2_. Find Bregma and Lambda on the surface of the skull in the eyepiece.

**[?TROUBLESHOOTING]**

24 Location. Location procedure follows the protocol published previously (Long* et al.* 2021). Details are as following.

(A) *X*-axis leveling

i. First, make a rough adjustment with the naked eye;

ii. Move and position the syringe needle to touch the Bregma on the surface of the skull, and reset the *x*, *y*, and *z* values of the desktop digital display to zero.

iii. Move the syringe needle to the right to the point where *x* = 2.0, and record *z*_1_ value; move the electrode to the left to the point where *x* = –2.0, and record *z*_2_ value. Leveling the left and right of the mouse brain to make |*z*_1_–*z*_2_| ≤ 0.03.

iv. After every adjustment of the position of the mouse brain, repeat Steps ii and iii.

**[TIP]** Before leveling, poke the skull with tweezers to make sure it will not move.

(B) *Y*-axis leveling

i. Move the syringe needle from Bregma (0, 0, 0) to Lambda, and record the *y*_L_ (Lambda of mature mice should be (0, –4.2, *z*_L_)) theoretically. If the mouse is retarded, then *y* < 4.2, the injection position should be adjusted in the same proportion, that is (*x*, *y*, *z*) × *y*_L_ /4.2.

ii. When the electrode is moved from Bregma to Lambda, recording the *z*_L_ (*z* value of Lambda). Leveling the *Y*-axis direction of the mouse brain to make |0–*z*_L_| ≤ 0.03.

iii. After every adjustment of the position of the mouse brain, repeat Steps (A) and (B).

25 Injection

(A) Locate the target brain area according to the *x*, *y*, *z* position of the target brain area, for example, dSTR (2.0, ±0.2, –2.6), and mark it with a marker.

(B) Drill a hole at the marking site with a cranial drill, drill once and align the electrode to see if the position is correct, repeat three times. Then drill for 2 s and stop for 1 s until the drill succeeds.

**[TIP]** Drill the hole vertically as much as possible; a little cerebrospinal fluid will leak out after drilling and wipe it clean, avoid bleeding; if both hemispheres are injected, drill both sides.

**[?TROUBLESHOOTING]**

(C) Calculate the dosage of α-syn PFFs (2 µg/µL) according to the weight of each mouse (0.2 µg/g), and suck the sample into the syringe with a micro syringe pump (parameters: set up, total volume: *n* µL, speed rate: 1 µL/min, mode: withdraw).

(D) On the surface of the hole drilled, set *z* to 0 and drop the electrode to the target position. Set the parameters of the micro syringe pump. Volume: 0.2 µL, rate: 0.5 µL/min, mode: infuse and start; then adjust to 0.2 µL/min, *n* – 0.2 µL.

**[CRITICAL STEP]** After injection, keep the micro syringe still for 5–6 min to prevent α-syn PFFs/PBS overflowing.

**[?TROUBLESHOOTING]**

26 Stitching. First, wipe the brain bones with PBS. Stitch the ends firstly, and then sew the middle. Finally, apply antibiotics to the wound and wipe off the eye ointment.

**[?TROUBLESHOOTING]**

27 Transfer the mice into a recovery cage under a warming lamp until mice wake up.

#### Behavioral tests [TIMING 12–14 d]

After injection, leave the mice under the same living conditions for three months.

#### Handle [TIMING 0.5–1 h for 24 mice/d, 3 d]

28 Put the mice on the palm for 1 min one by one. If the mice climb along the arm, put them back to the palm. The expected result of handling is that every mouse stays still on the palm.

**[CRITICAL STEP]** Before each behavioral test, transfer mice to the behavioral room at least 1 h in advance to adapt to the testing environment.

#### Open field test [TIMING 2–3 h for 24 mice]

29 Adjust and test the instruments. For example, fix the camera on the ceiling and setting the light inside each chamber at 30–35 lux.

30 Put each mouse in the middle of the chamber (40 cm × 40 cm) for 10 min. Their activities are monitored and recorded by the camera and analyzed by EthoVision XT (Noldus11.5).

**[TIP]** Researchers need to leave the room to reduce human interferences. Change the chamber of the same group and clean the chamber with 75% alcohol between each trial.

**REST** [TIMING 1 d]

#### Rotarod training [TIMING 3 h/d for 24 mice, 3–5 d]

31 Train the mice twice for 2 min with constant speed, 4 r/min. The 3^rd^ trial goes on with accelerating speed from 0 to 40 r/min in 1.5 min, total of 2 min. Record the total duration of the mouse on the rotating rod. There is a 3-min interval between each trial. All mice are trained for 3–5 d consecutive days until most mice perform well.

**REST** [TIMING 1 d]

32 Mice receive two tests continuously on the rotarod expediting from 0 to 40 r/min in 1.5 min, then keep at 40 r/min for another 3.5 min. Record the total duration of the mouse on the rotating rod.

**[TIP]** Clean the rotarod with 75% alcohol between each trial. At least three consecutive or discontinuous turns of the mouse following the rotarod are deemed as landing.

**REST **[TIMING 1 d]

#### Pole test [TIMING 3 h for 24 mice]

33 Climbing. Place each mouse on the top of the metal rod (length: 50 cm; OD: 1 cm) that is wrapped with medical tape. Guide the mouse head facing downwards. Train all mice to climb down the rod once before testing for five times continuously and record the climb-down duration time.

**[TIP]** No more than 60 s for each test. When the climbing test is over, put all mice back to their homecages to rest for more than 30 min.

34 Turning. Place the mice on the top of the rod with their heads facing upwards and record the time for the mice turning their heads downwards, repeat five times.

### Histopathological studies [TIMING 17 d]

#### Sectioning [TIMING 5 d]

35 Perfusion. Anesthetize mice with isoflurane vapor and transcranially perfuse them with ice-cold PBS (20 mL) followed by 4% PFA (40 mL) slowly. Remove the whole brain and keep them in 4% PFA overnight.

**[?TROUBLESHOOTING]**

36 Dehydration. Transfer brains to 20% sucrose for dehydration, at least 24 h. Until brains sink to the bottom of the 15 mL centrifuge tubes, transfer them to 30% sucrose, overnight. When brains sink to the bottom again, transfer them to 30% sucrose again. After dehydration, put all brains into embedding boxes filled with optimal cutting temperature compound (OCT) and rapidly freeze at –80 °C.

**[?TROUBLESHOOTING]**

37 Cryosection. Serially section all brains into 30-μm slices with a cryostat microtome (Leica). Collect and classify brain slices, and keep them in cryoprotectant solution at –20 °C.

#### Immunofluorescence staining [TIMING 2 d]

38 Washing. Wash all slices with PBS for three times, 10 min/time. Keep them at 4 °C for storage.

39 Blocking. Block non-specific sites with 5% H_2_O_2_, 5% normal goat serum and 0.1% Triton X-100 in PBS at RT for 2 h.

40 Primary antibody incubation. Brain slices are incubated with the primary antibody diluted with the blocking solution containing phospho-α-syn (S129) monoclonal rabbit antibody (1:250, abcam, ab51253) and DAT (dopamine transporter) monoclonal rat antibody (1:500, abcam, ab5990) at 4 °C overnight, shaking.

**[TIP]** The ratio of antibodies used can be optimized.

41 Washing. Brain slices are then rinsed in PBST, 10 min/time for three times and once more with PBS.

42 Secondary antibody incubation. All brain slices are incubated with the secondary antibodies diluted with the blocking solution containing goat anti-rabbit Alexa Fluor 568 (1:1000, abcam, ab175471) and goat anti-rat Alexa Fluor 488 (1:1000, abcam, ab150157) for 2 h at RT.

**[TIP]** The ratio of antibodies used can be optimized.

43 Washing. Brain slices are then rinsed in PBST, 10 min/time for three times and once more with PBS.

44 The slices are mounted on glass slides with Prolong gold antifade reagent to preserve fluorescence signal. Store at 4 °C.

#### Confocal imaging [TIMING 10 d]

45 Imaging. A spinning disk fluorescence confocal microscope is required to capture fluorescence images. Two kinds of selective lens patterns are applied, 20× (NA = 0.75) air objective for SN and 40× (NA = 1.25) water immersion objective for STR. Lasers of 630, 561 and 488 nm are used to excite Alexa Flour 647, 568 and 488, respectively and sequentially.

46 Image analysis and statistical analysis.

**[?TROUBLESHOOTING]**

Troubleshooting advices can be found in Table 1.

**Table 1:** Troubleshooting table

Step	Problem observed	Possible reason	Solutions
9	Data of CCK-8 assay are of poor reproducibility	Improper operation or too few sample repeats	Set up more sample repeats
10	Primary neuron cultured in plates are polluted	(1) The plate is polluted (2) Brain tissue is contaminated during dissection	(1) Irradiate plates with UV (2) Add penicillin-streptomycin to HBSS buffer
13	Poor digestion of brain tissue	Insufficient activation of papain	Activate papain until the solution is translucent
17	Primary neuron died after treated by PFFs	Primary neuron is contaminated by PFFs	Washing α-syn fibrils with PBS before sonication
19	Cells left on coverslips are too few	Cells are washed away	Rinse cells gently
23	Bregma and Lambda are not clearly visible	Meninges on the skull	Corrode the menings with 5% H_2_O_2_
25	Bleed when drilling holes on skull	Injured brain tissue when drilling	Observe carefully while drilling
25	No sample comes out of the electrode	The electrode is blocked by PFFs	Gently grip the electrode tip with tweezers
26	The surgical thread fell off after the operation	Improper operation	Operation properly or use bio fibrin glue
35	Blood vessel interference during fluorescence imaging	Poor perfusion effect	Operate perfusion properly
36	Brain slices are easily broken in PBS	Incomplete dehydration	Make sure the complete dehydration

## ANTICIPATED RESULTS

**Figure 1:**
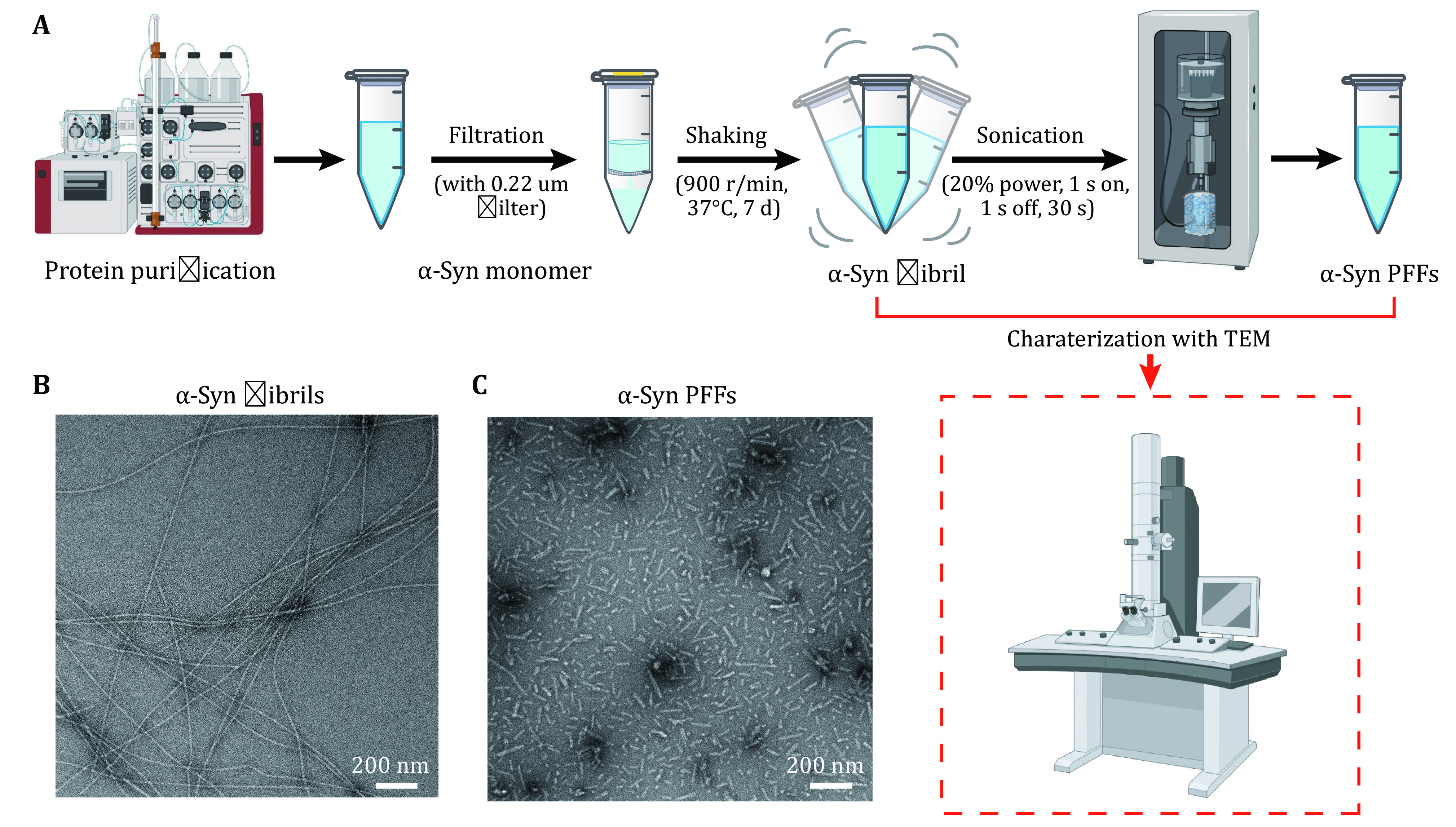
Preparation and characterization of α-syn PFFs. **A** Steps of PFFs preparation. The key step is sonication, which fragments long fibrils into short fibrils with similar lengths. **B**, **C** Negative-staining TEM images of α-syn fibrils (**B**) and α-syn PFFs (**C**). Scale bar, 200 nm

1 Following the protocol, we first prepared α-syn PFFs which were characterized by TEM. Figure 1A shows the operating process. The homogeneous and unbranched fibrils were imaged by TEM (Fig. 1B). α-Syn fibrils were sonicated into α-syn PFFs, which were well dispersed and uniform in size (Fig. 1C).

**Figure 2:**
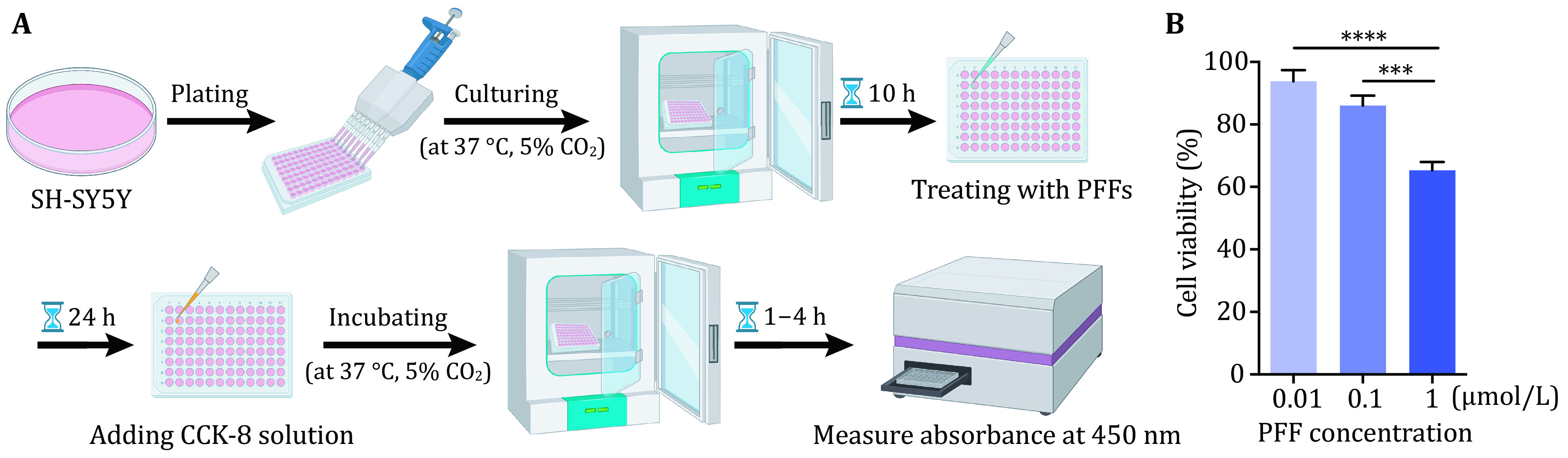
SH-SY5Y cell viability assay. **A** Schematic diagram of cell viability assay. **B** Demonstration of the result of cell viability after treatment with α-syn PFFs analyzed by GraphPad Prism 6.01. Data shown are mean ± SD, *n* = 3 independent samples. The level of significance was set as ***p < 0.001, ****p < 0.0001

**Figure 3:**
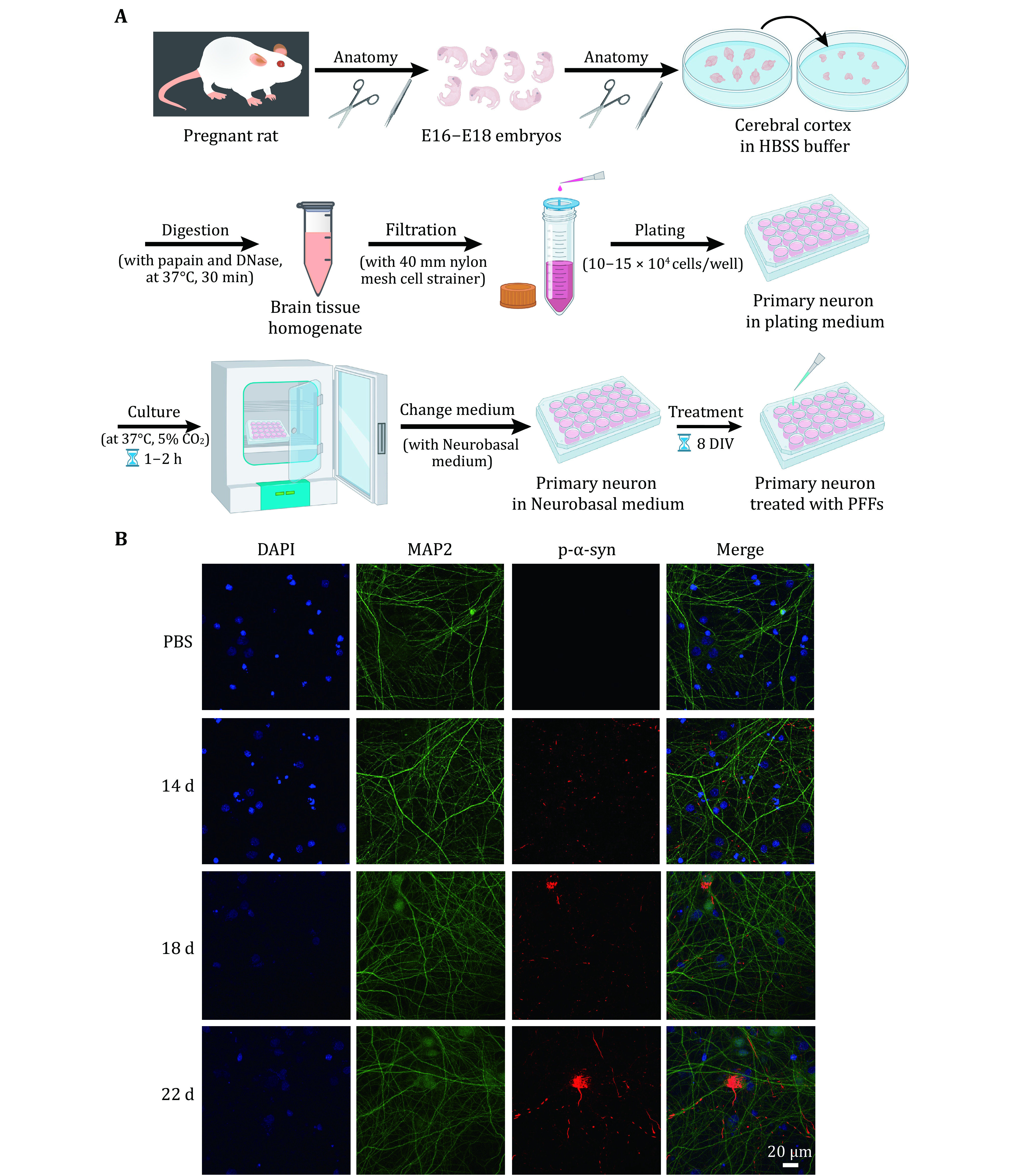
Rat primary neuron model for testing the transmission and propagation of exogenous α-syn PFFs in neurons. **A** Schematic diagram of primary neuron culturing. On the 8^th^ day of neuron cultured *in vitro* (8 DIV), treat the neurons with α-syn PFFs and PBS (control), respectively. **B** Representative immunofluorescence images of primary neurons treated for 14, 18 and 22 d, respectively. Antibodies were used to mark DAPI (blue), MAP2 (green) and p-α-syn (red). Scale bar, 20 μm

2 The SH-SY5Y cells cultured in a 96-well plate were treated with sterilized α-syn PFFs with final concentrations of 0, 0.01, 0.1, 1 µmol/L for 24 h (Fig. 2A). The result showed that the cell viability decreased with increasing concentrations of PFFs (Fig. 2B). Next, we cultured rat primary neurons and treated them with sterilized α-syn PFFs at 8 DIV with a final concentration of 100 nmol/L (Fig. 3A). Then the neurons were further incubated until 22 DIV, 26 DIV, 30 DIV and so on. Endogenous α-syn aggregates were induced by the addition of α-syn PFFs with increasing amounts over time in primary neurons (Fig. 3B). These results demonstrate the transmission of α-syn PFFs from medium to cell interior and the propagation of α-syn PFFs in cells.

**Figure 4:**
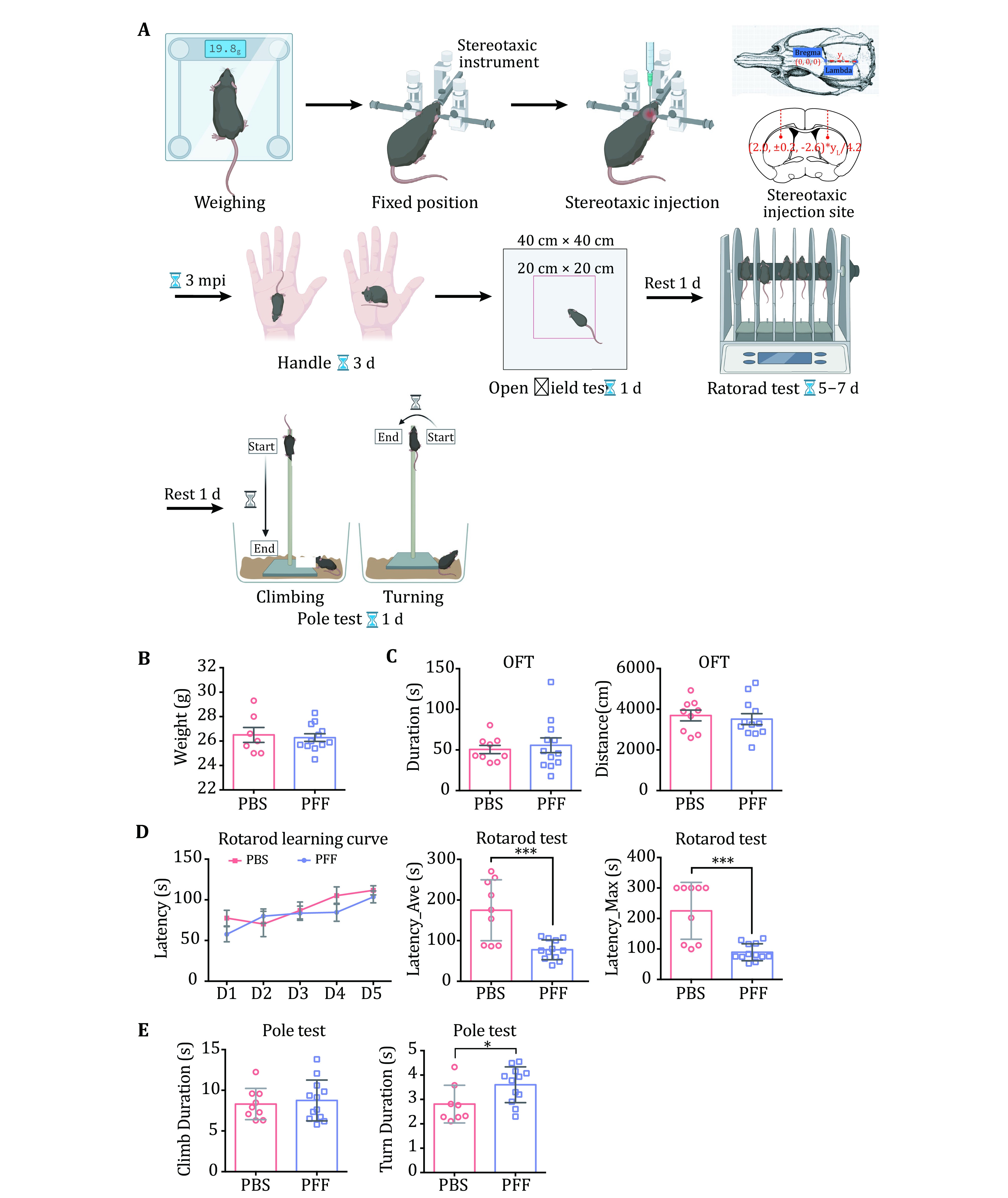
Establishment of PD-related mouse model and behavioral tests. **A** Stereotaxic injection and an overview of behavioral tests. The dose for injection is determined based on the body weight of individual mice. Three kinds of behavioral tests are illustrated including open field test (OFT), rotarod and pole tests. Experimental data are demonstrated in B–E. **B** Body weight of mice after PFF injection. **C** OFT. Left, duration of OFT refers to the time that each mouse stays in the center area (20 cm × 20 cm). Right, distance statistics refer to the distance traveled by each mouse. **D** Rotarod tests. The normalized learning curve is on the left. The average time of the final rotarod test is in the middle. The maximum time of the final rotarod test is on the right. **E** Pole test. The two graphs show the time needed for climbing up-to-bottom (left) and turning up-side-down (right) in the pole test. Data shown are mean ± SD, the level of significance was set as *p < 0.05; ***p < 0.001

**Figure 5:**
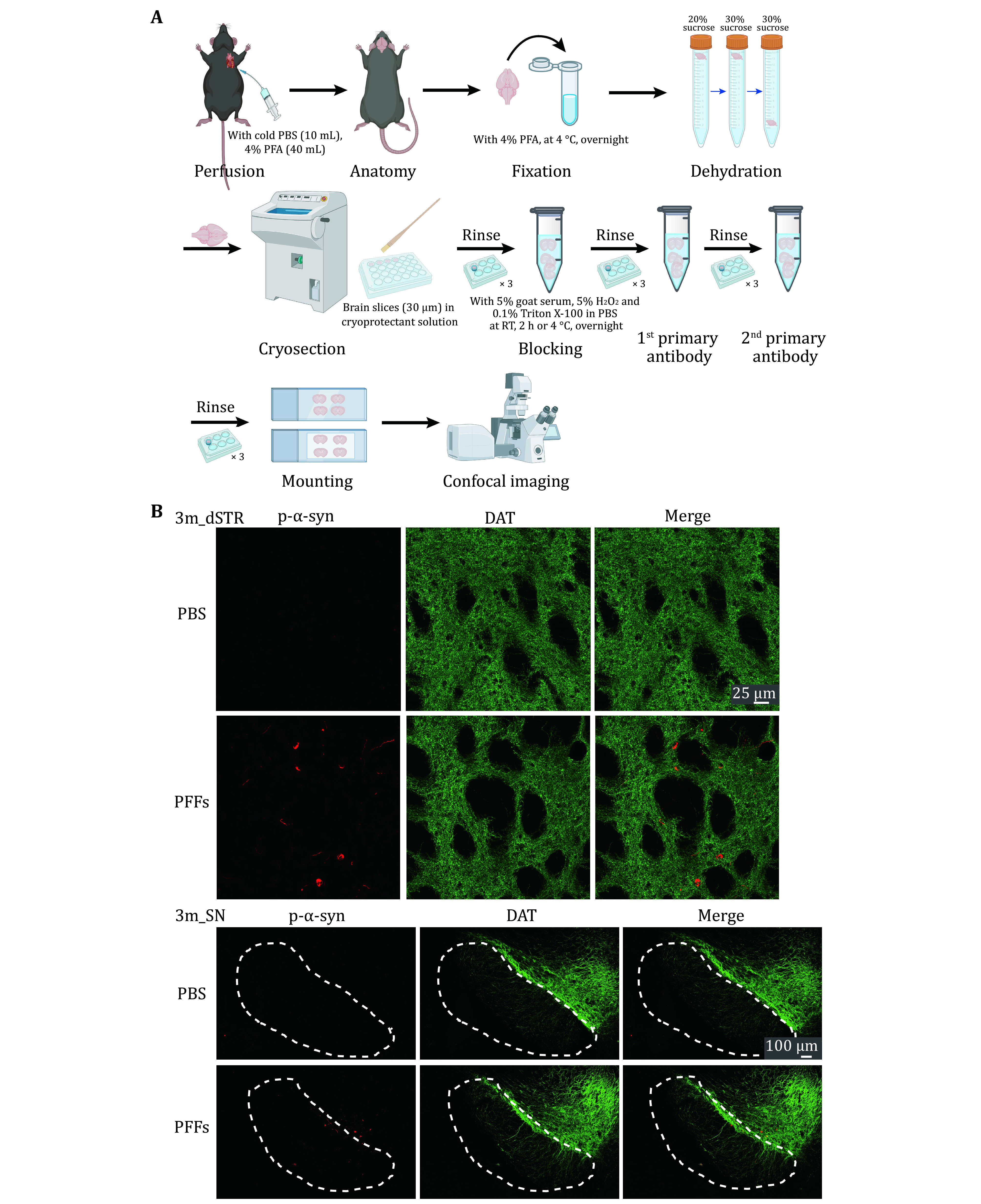
Immunofluorescence staining of PD-related mouse brain. **A** Workflow of immunofluorescence staining of PD-related mouse brain. **B** Representative data of the immunostaining. Images of dSTR (top) and SN (bottom) 3-month post inject are shown respectively. Antibodies were used to mark p-α-syn (red) and DAT (green)

3 To investigate the neuropathology induced by α-syn PFFs *in vivo*, PFFs were bilaterally inoculated into the dorsal striatum of WT C57BL/6J mice. Motor performances were performed at 3 mpi (Fig. 4A). The data of OFT showed that there was no difference between α-syn PFFs-injected and PBS-injected mice (Fig. 4C). However, mice inoculated with α-syn PFFs performed worse than PBS-injected control mice in the rotarod and pole test (Fig. 4D, E). We further cryo-sectioned the brain tissues of the mice into 30-µm coronal slices and examined α-syn pathology at this time point by immunofluorescence staining (Fig. 5A). Images of dSTR showed obvious α-syn aggregates in the group of PFFs-injected mice (Fig. 5B). Furthermore, the aggregates were also observed in the substantia nigra (SN) (Fig. 5B). These results demonstrate that α-syn PFFs efficiently induced endogenous aggregates and α-syn pathology *in vivo*.

## MATERIALS

### Reagents

• Source materials, *e*.*g*., tissues and organs for primary neuron culture, are from Sprague-Dawley rats. Mice for establishing PD models are C57 BL/6J mice.

**[CAUTION!]** All experiments using animals must be carried out according to relevant governmental and institutional regulatory guidelines.

• NaCl (Sangon Biotech, cat. no. A100241-0500)

• KCl (Sangon Biotech, cat. no. A501159-0500)

• HCl (Hushi, cat. no. 10011018)

**[CAUTION!]** HCl is acutely toxic, unstable and explosive. Avoid flames and handle in a fume hood. Disposal must be in accordance with local regulations.

• Ethanol Absolute (Hushi, cat. no. 100092680)

• 75% alcohol (Hushi, cat. no. 801769610)

• Sucrose (Sangon Biotech, cat. no. A502792-0500)

• Boric acid (Sigma Aldrich, cat. no. B-0252)

• Borax (Sigma Aldrich, cat. no. B-9876)

• B-27 Supplement (50×) (Gibco, cat. no. 17504044)

• GlutaMAX (Gibco, cat. no. 35050061)

• EDTA (Sangon Biotech, cat. no. A100105-0500)

• PBS (Sangon Biotech, cat. no. E607008-0500)

• Tris (Sangon Biotech, cat. no. A501492-0500)

• NaN_3_ (Sigma Aldrich, cat. no. S2002)

• Glycerol (Hushi, cat. no. 10010618)

• 16% paraformaldehyde (Shaoxin Biotech, cat. no. SX20197)

**[CAUTION!]** 16% PFA is toxic and volatile with a pungent odor. Make sure to wears a mask and operate in the fume hoods. Disposal must be in accordance with local regulations.

• β-Mercaptoethanol (Sigma Aldrich, cat. no. M6250-500ML)

**[CAUTION!]** β-Mercaptoethanol is volatile with a pungent odor. Make sure that wear a mask and operate in the fume hood during experiments. Dispose of in accordance with local regulations.

• Penicillin streptomycin (PS) (Gibco, cat. no. 15140122)

• Triton X-100 (Sangon Biotech, cat. no. A600198-0500)

• HEPES (BBI Life Sciences, cat. no. A600264-0250)

• OCT Tissue Freezing Medium (Leica, cat. no. 14020108926)

• PDL (Sigma Aldrich, cat. no. P0899-10MG)

• Hank’s Balanced Salt Solution (HBSS) (Gibco, REF 14175-095)

• DMEM (Gibco, cat. no. 11995065)

• Neurobasal medium (Gibco, cat. no. 21103049)

• Goat Serum Albumin (Gibco, cat. no. 16210072)

• Bovine Serum Albumin (Gibco, cat. no. 10099-141)

• Isoflurane (Reward Biotech, cat. no. R510-22)

**[CAUTION!]** Isoflurane is volatile with a pungent odor. Make sure to wear a mask and operate in the fume hood. Disposal must be in accordance with local regulations.

• Phospho-α-synuclein (S129) monoclonal rabbit antibody (Abcam, cat. no. ab51253)

• DAT monoclonal rat antibody (Abcam, cat. no. ab5990)

• NeuN polyclonal chicken antibody (Millipore, cat. no. ABN91)

• Goat anti-rabbit IgG Alexa Fluor 568 (Abcam, cat. no. ab175471)

• Goat anti-rat IgG Alexa Fluor 488 (Abcam, cat. no. ab150157)

• Goat anti-chicken Alexa Fluor 488 (Thermo Fisher, cat. no. A-11039)

• Papain (Sangon Biotech, cat. no. A003124)

• ProLong™ Gold Antifade Mountant with DAPI (Thermo Fisher, P36941)

• ProLong Gold Antifade reagent (Thermo Fisher, P36930)

• CCK-8 kit (Yeasen, 40203ES60)

• DNase I (Roche, cat. no. 10104159001)

• Trypsin-EDTA (0.25%), phenol red (Gibco, cat. no. 25200072)

### Reagent setup

• 3 mol/L KCl stock. Dissolve 223.65 g KCl in 900 mL deionized water and stir until it is dissolved. Bring the volume to 1 L and filter-sterilize the solution. This solution can be stored at room temperature.

• 1 mol/L Tris (pH 7.5) stock. Dissolve 121.14 g Tris in 900 mL deionized water and stir until it is dissolved. Adjust the pH to 7.5 with HCl. Bring the volume to 1 L and filter-sterilize the solution. This solution can be stored at room temperature.

• 500 mmol/L EDTA Stock. Dissolve 186.12 g EDTA in 900 mL deionized water and stir until dissolved. Bring the volume to 1 L and filter-sterilize the solution. This solution can be stored at room temperature.

• Sucrose (20%). Dissolve 100 g sucrose in 400 mL deionized water and stir until most of it is dissolved. Bring the volume to 500 mL and stir until fully dissolved. This solution can be stored at 4 °C for three months.

• Sucrose (30%). Dissolve 150 g sucrose in 400 mL deionized water and stir until most of it is dissolved. Bring the volume to 500 mL and stir until fully dissolved. This solution can be stored at 4 °C for three months.

• Borate buffer (0.1 mol/L). Dissolve 1.24 g boric acid and 1.90 g borax in 400 mL deionized water and stir until most of it is dissolved. Adjust the pH to 8.5 with NaOH. Bring the volume to 500 mL and filter-sterilize the solution. This solution can be stored at 4 °C.

• Blocking solution (for mouse brain slices). 5% normal goat serum and 0.1% Triton X-100 in PBS.

• Fixation solution for primary neuron culture. Add 4% PFA and 4% sucrose to PBS.

• Permeabilization solution. Add 0.15% Triton X-100 to PBS.

• PBST. Add 0.1% Triton X-100 to PBS.

• Cryoprotectant solution. 30% (*w*/*v*) sucrose, 20% (*w*/*v*) ethylene glycol, 50% (*w*/*v*) PBS. This solution can be stored at –20 °C for three months.

• Plating medium. Add 10% (*w*/*v*) FBS, 1% (*w*/*v*) PS to 500 mL DMEM medium. Before each use, heat at 37 °C in advance.

• Neurobasal medium. Combine 40 mL Neurobasal medium, 800 µL B27 from a 50× stock, 100 µL GlutaMAX from a 100× stock and 400 µL PS (1,000 U/mL penicillin and 1,000 µg/mL streptomycin). Filter-sterilize the solution and store it at 4 °C for no longer than three weeks. Before each use, heat at 37 °C in advance.

• HBSS buffer. Add 5–10 mL 1 mol/L HEPES to 500 mL HBSS.

• PDL buffer. Add PDL to borate buffer with a final concentration of 0.01 mg/mL.

• Papain buffer. 1.1 mmol/L EDTA, 0.067 mmol/L β-mercaptoethanol and 5.5 mmol/L cysteine-HCl.

• DNase. Dissolve DNase to 10 U/mL in HBSS (above); divide it into aliquots and store them at –20 °C.

### EQUIPMENTS

• Microscope Cover Glasses (Fisherbrand, cat. no. 1254580)

• Adhesion microscope slides (Citotest, cat. no. 188105)

• Embedding box (Surui)

• 0.22-μm centrifugal filter (Millipore, cat. no. SLGP033NS)

• 1.5-mL microtube (Axygen, cat. no. MCT-150-C)

• 15-mL Centrifuge tube (YUEYI, cat. no. YB0019-15)

• 50-mL Centrifuge tube (YUEYI, cat. no. YB-50D)

• 6-well plate (Corning, cat. no. 3516)

• 24-well plate (Corning, cat. no. 3524)

• 96-well (Corning, cat. no. 3590)

• Pipettes: 25, 10, 5 and 1mL

• Pipettor (Eppendorf, Easypet 3)

• −80 °C freezer (Thermo Fisher Scientific)

• Probe tip sonicator (Xinyi, JY92-IIN)

• Ultrasonic cleaner (Kunshan Shumei, KQ5200E)

• Benchtop centrifuge for 1.5 mL tube (up to 15,000 *g*)

• Thermomixer with block for 1.5 mL tube (Eppendorf)

• Fume hood (Shanghai TL Chemical)

• Water bath, 37 °C

• Transmission electron microscope (FEI, Tecnai, 120 kV TWIN)

• Stereotaxic apparatus (Qianaoxingke, SH-01A/B)

• Anesthesia apparatus (Qianaoxingke，IA 5）

• Oxygen generator (Qianaoxingke, XY-3/3C/3S)

• Microsyringe pump (Beijing Xianyi Biotech, 53311)

• Glow discharge cleaning system (Ted Pella, PELCO easiGlow)

• Nanodrop 2000c (Thermo Fisher Scientific)

• Miniature hand-held cranial drill (Reward)

• Benchtop refrigerated centrifuge (Beckman Coulter, Microfuge 11)

• Microprocessor-controlled mini benchtop centrifuge (Beckman Coulter, Microfuge 16)

• High-speed centrifuge (Beckman Coulter, Avanti j-26S XP)

• Water-jacket Thermostatic Incubator (Thermo Scientific)

• EthoVision XT (Noldus11.5)

• Rotarod system (Sans, SA102)

• Cryostat microtome (Leica, CM 1860)

• Fluoroskan Ascent microplate reader (Thermo Scientific, Varioskan Flash)

• Laser scanning confocal microscope (Leica, TCS SP8)

• Spinning disk fluorescence confocal microscope (Andor, Drangonfly)

## Conflict of interest

Houfang Long, Shuyi Zeng and Dan Li declare that they have no conflict of interest.
